# IgG4-Related Sclerosing Cholangitis, a Rare Differential Diagnosis for a Common Clinical Presentation: A Case Report

**DOI:** 10.1159/crg/abwag011

**Published:** 2026-09-10

**Authors:** Tiago Alexandre Ventura Antunes, Diogo Feijó, João Maldonado, Maria João Amaral, Marco Serôdio, Catarina Melo, J. Guilherme Tralhão

**Affiliations:** a Department of General Surgery, Unidade Local de Saúde de Coimbra, Coimbra, Portugal; b Department of Gastroenterology, Unidade Local de Saúde de Coimbra, Coimbra, Portugal; c Department of Pathology, Unidade Local de Saúde de Coimbra, Coimbra, Portugal; d Faculty of Medicine, University of Coimbra, Coimbra, Portugal; e Biophysics Institute, Faculty of Medicine, University of Coimbra, Coimbra, Portugal; f Coimbra Institute for Clinical and Biomedical Research (iCBR) area of Environment, Genetics and Oncobiology (CIMAGO), Faculty of Medicine, University of Coimbra, Coimbra, Portugal

**Keywords:** Jaundice, Differential diagnosis, Inflammatory pseudotumor, Cholangiocarcinoma

## Abstract

**Introduction:**

Immunoglobulin G4-related sclerosing cholangitis (IgG4-SC) is a rare immune-mediated fibroinflammatory disease that represents a significant diagnostic challenge, as it may closely mimic malignant biliary neoplasms, particularly cholangiocarcinoma (CCA).

**Case Presentation:**

This report describes a 71-year-old man with a history of cholecystectomy for acute calculous cholecystitis who presented with new-onset obstructive jaundice. Magnetic resonance cholangiopancreatography demonstrated parietal thickening of the common hepatic duct at the confluence of the right and left hepatic ducts, associated with diffusion restriction and intrahepatic biliary dilatation. Serum CA 19-9 was markedly elevated, even after biliary drainage and endoluminal biopsies were inconclusive. Given the strong suspicion of perihilar CCA, the patient underwent right hepatectomy extended to segment 1 with extrahepatic bile duct resection and Roux-en-Y hepaticojejunostomy. Definitive histopathological examination revealed IgG4-SC involving both extrahepatic and intrahepatic bile ducts, without evidence of malignancy.

**Conclusion:**

Isolated IgG4-SC remains a major diagnostic challenge because its clinical, biochemical, endoscopic, and radiological features may closely mimic CCA. This case highlights the limitations of current preoperative diagnostic tools and emphasizes the importance of considering IgG4-SC in the differential diagnosis of isolated biliary strictures. Although earlier assessment of serum IgG4 may broaden the diagnostic workup, definitive diagnosis may still only be achieved on histopathological examination. When malignancy cannot be confidently excluded, surgical resection remains an appropriate and often unavoidable management strategy.

## Plain Language Summary

This report describes a rare condition that can closely resemble bile duct cancer. The condition, called IgG4-related sclerosing cholangitis, is an inflammatory disease in which the body’s immune system causes swelling and narrowing of the bile ducts. Bile ducts are tubes that carry bile from the liver to the intestine to help digest food. A 71-year-old man developed jaundice, a yellowing of the skin and eyes caused by blockage of bile flow. Imaging tests suggested a tumor in the bile ducts, and blood tests showed high levels of a marker often linked to cancer. Because cancer could not be ruled out, the patient underwent major liver surgery. However, examination of the removed tissue showed that the cause was not cancer but the inflammatory condition described above. This case shows how difficult it can be to distinguish between this inflammatory disease and cancer before surgery. The two conditions can look very similar on scans and tests. Recognizing this disease is important because it can often be treated with medication instead of surgery. Testing for specific immune markers in the blood may help doctors consider this diagnosis earlier. Greater awareness of this condition may help avoid unnecessary major operations and allow patients to receive the most appropriate treatment.

## Introduction

Immunoglobulin G4-related sclerosing cholangitis (IgG4-SC) is an immune-mediated fibroinflammatory disease characterized by infiltration of intrahepatic and extrahepatic bile ducts by IgG4-positive lymphoplasmacytic cells [1, 2]. It belongs to the spectrum of IgG4-related disease and is associated with type 1 autoimmune pancreatitis in up to 92% of cases, whereas isolated biliary involvement is uncommon [3, 4]. Other manifestations of IgG4-related disease are summarized in Table 1 [3, 5].

**Table 1.** IgG4-related diseases

Hypertrophic pachymeningitis	Constrictive pericarditis
Hypophysitis	Large vessel arteritis
Dacryocysitis	Mediastinal and retroperitoneal fibrosis
Orbital myositis	Autoimmune pancreatitis (type I)
Sialadenitis	Sclerosing cholangitis
Nasal rhinosinusitis	Tubulointerstitial nephritis and membranous glomerulonephropathy
Riedel’s thyroiditis	Prostatitis
Pulmonary and pleural interstitial disease	Lymphadenopathies

The estimated incidence of IgG4-SC is approximately 1.4 per 100,000 population, with a marked male predominance, particularly between 60 and 70 years of age [2, 6]. Symptomatic patients typically present with progressive obstructive jaundice that may involve any segment of the biliary tree. Advanced disease may progress to secondary biliary cirrhosis and ultimately require liver transplantation [6].

Diagnosis relies on a combination of elevated serum IgG4 concentrations, characteristic imaging findings of multifocal or diffuse biliary strictures and histopathological evidence of IgG4-rich lymphoplasmacytic infiltrate (>10 cells per high-power field), storiform fibrosis, and obliterative phlebitis [2, 4, 7]. However, diagnosis can be particularly challenging in cases presenting with a solitary biliary stricture that mimics malignancy [8]. We report a rare case of isolated perihilar stenosis due to IgG4-SC initially diagnosed as perihilar cholangiocarcinoma (CCA).

## Case Presentation

A 71-year-old Caucasian man was referred to the surgical outpatient clinic for evaluation of progressive obstructive jaundice associated with generalized pruritus and mild right upper quadrant pain radiating to the back. His medical history included laparoscopic cholecystectomy for acute calculous cholecystitis, arterial hypertension, type 2 diabetes mellitus, dyslipidemia, and AL-type amyloidosis involving the stomach and duodenal bulb under hematological surveillance. Occupational history exposed long-term employment in railway infrastructure maintenance. Physical examination revealed scleral icterus and mild jaundice. Abdominal palpation revealed mild tenderness in the right upper quadrant without palpable masses.

Laboratory tests demonstrated a marked cholestatic pattern of liver injury, with elevated liver enzymes including aspartate aminotransferase (405 U/L), alanine aminotransferase (523 U/L), alkaline phosphatase (ALP; 247 U/L), and gamma-glutamyl transferase (GGT; 1033 U/L). Total bilirubin was elevated at 5.4 mg/dL (normal 0.3–1.2), with a direct fraction of 3.3 mg/dL (normal <0.5), consistent with obstructive cholestasis. Abdominal computed tomography did not reveal clear biliary dilatation or an obstructing lesion. Further evaluation was therefore undertaken, including serological testing, autoimmune screening, protein electrophoresis, and magnetic resonance cholangiopancreatography. Serological tests confirmed immunity to hepatitis A virus, cytomegalovirus, and Epstein-Barr virus (Table 2), while autoimmune screening and immunofixation studies were negative (Tables 3, 4). Protein electrophoresis showed a monoclonal lambda light-chain band consistent with known AL amyloidosis (Fig. 1; Table 5) [9].

**Table 2.** Serologies

Anti-HAV antibody, IgM	Negative
Anti-HAV antibody, IgG	Positive, titer 9.36 (Neg <1.00)
Anti-HBsAg antibody	Negative
AgHBs	Negative
Anti-HBc antibody	Negative
Anti-HCV antibody	Negative
Anti-CMV antibody, IgM	Negative
Anti-CMV antibody, IgG	Positive, titer 30.00 (Neg <4.00)
EBV-VCA antibody, IgM	Negative
EBV-VCA antibody, IgG	Positive, titer 2.53 (Neg <0.09)
EBV-EBNA antibody, IgG	Positive, titer 5.95 (Neg <0.09)
Anti-toxoplasma antibody, IgM	Negative
Anti-toxoplasma antibody, IgG	Positive, titer 12.00 (Neg <0.55)
Anti-*Borrelia burg*. antibody, IgM	Negative
Anti-*Borrelia burg*. antibody, IgG	Negative
Anti-HIV 1/2 antibody	Negative
Anti-*Treponema pallidum* antibody	Negative

HAV, hepatitis A virus; HBV, hepatitis B virus; HCV, hepatitis C virus; EBV, Epstein-Barr virus; HIV, human immunodeficiency viruses.

**Table 3.** Autoimmunity

Anti-nuclear and cytoplasmic antibody	Negative
Anti-neutrophil cytoplasm antibody	Negative
Anti-mitochondrial antibody	Negative
Anti-smooth muscle antibody (F-actin)	Negative
Anti-LKM antibody	Negative

**Table 4.** Immunofixation

Immunoglobulin G, g/L	14.80
Immunoglobulin A, g/L	1.54
Immunoglobulin M, g/L	3.89
Kappa light chains, g/L	12.70
Lambda light chains, g/L	7.37
Kappa/lambda ratio	1.72

Reference values: immunoglobulin G 7.51–15.60, immunoglobulin A 0.82–4.53, immunoglobulin M 0.46–3.04, kappa light chains 6.29–13.50, lambda light chains 3.13–7.23, kappa/lambda ratio 1.50–2.00.

**Fig. 1.**
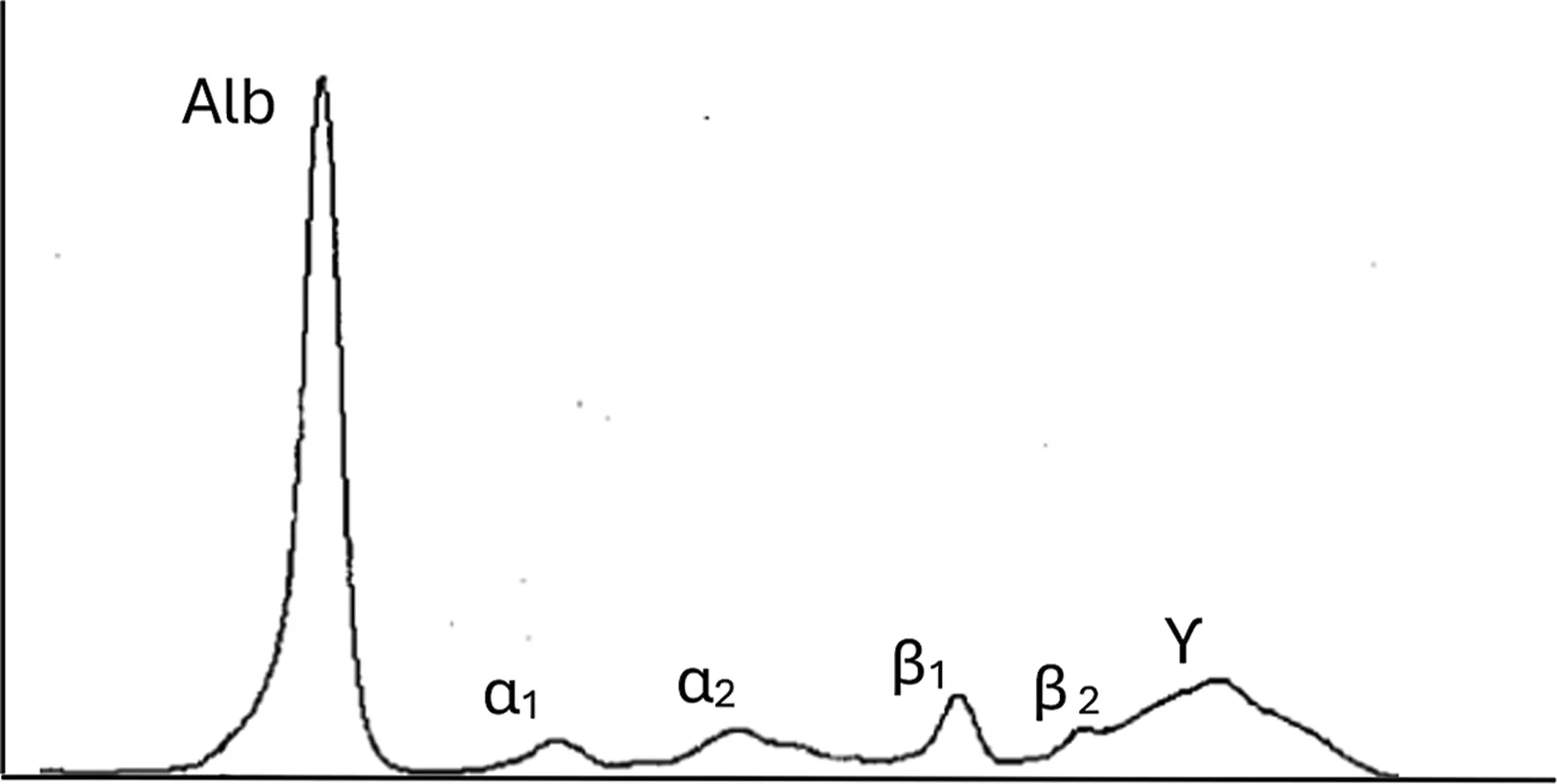
Protein electrophoresis showed the presence of a very discreet lambda monoclonal band.

**Table 5.** Protein electrophoresis

Albumin fraction, g/dL	3.37	51.9%
α1-fraction, g/dL	0.27	4.2%
α2-fraction, g/dL	0.64	9.8%
β1-fraction, g/dL	0.44	6.7%
β2-fraction, g/dL	0.32	5.0%
γ-fraction, g/dL	1.46	22.4%
Albumin/globulin ratio	1.08	

Reference values: albumin fraction 4.02–4.76, α1-fraction 0.21–0.35, α2-fraction 0.51–0.85, β1-fraction 0.34–0.52, β2-fraction 0.23–0.47, γ-fraction 0.80–1.35.

Magnetic resonance cholangiopancreatography demonstrated parietal thickening of the common hepatic duct at the confluence of the right and left hepatic ducts, associated with diffusion restriction and intrahepatic biliary dilatation, as well as multiple small lymph nodes in the hepatic hilum and celiac region (Fig. 2). Serum carcinoembryonic antigen levels were within normal limits, whereas CA 19-9 was elevated (391.6 U/mL). The case was discussed at a multidisciplinary hepatobiliary meeting, and imaging findings were considered highly suggestive of Bismuth-Corlette type II perihilar CCA involving the right hepatic artery, although inflammatory stenosis remained a differential diagnosis.

**Fig. 2.**
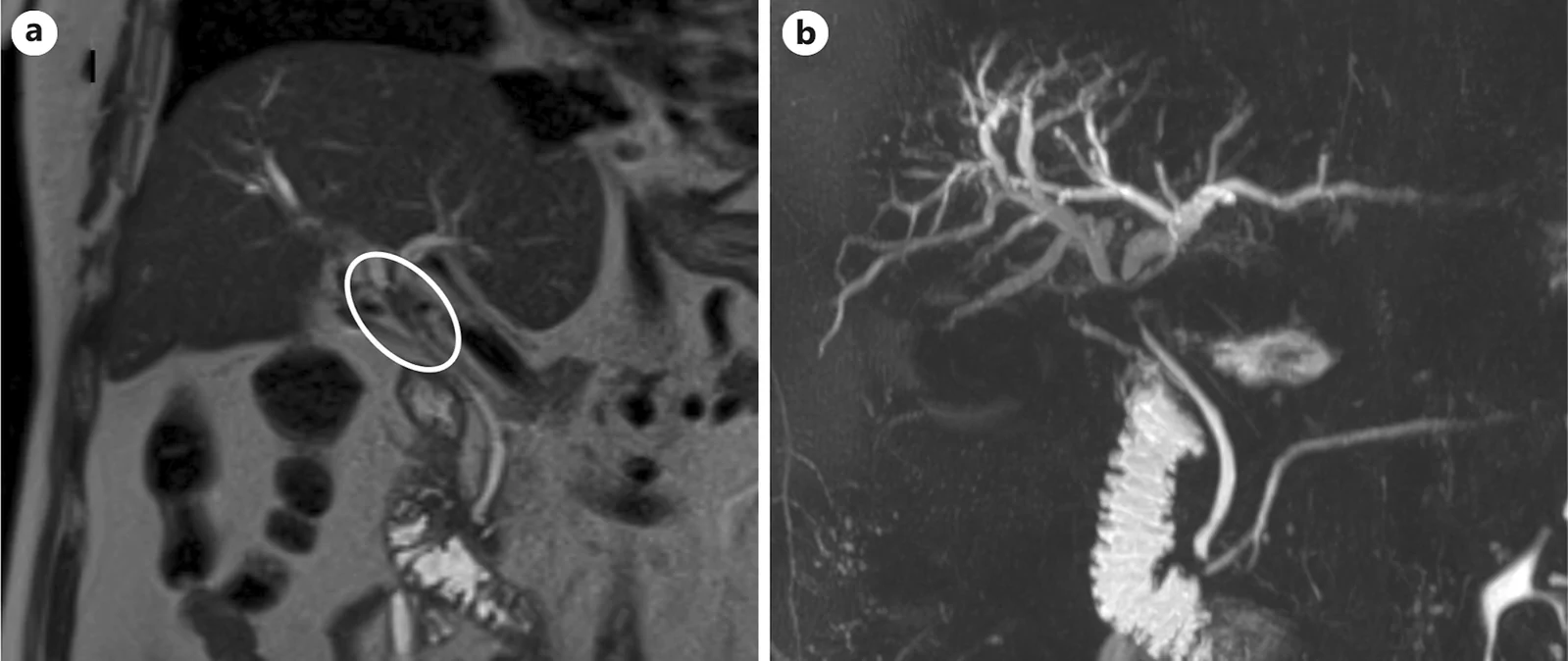
Cholangio-MRI showed a parietal thickening in the common hepatic duct, at the confluence of the right and left ducts, with diffuse restriction and dilatation of the intrahepatic bile ducts in T2 weighting coronal section (**a**) and 3D reconstruction (**b**).

Endoscopic retrograde cholangiopancreatography with cholangioscopy revealed a high-grade stenosis of the proximal common bile duct, with extensive irregular mucosa in the proximal common bile duct (Fig. 3). Targeted cholangioscopy-guided biopsies and brush cytology were obtained. A 10-Fr × 12 cm plastic biliary stent was placed, achieving satisfactory biliary drainage. Following drainage, liver biochemistry improved substantially, with aspartate aminotransferase decreasing from 405 U/L to 61 U/L, alanine aminotransferase from 523 U/L to 70 U/L, GGT from 1,033 U/L to 294 U/L, total bilirubin from 5.4 mg/dL to 1.2 mg/dL, and CA 19-9 remained elevated (273 U/mL after drainage). Targeted cholangioscopy-guided biopsies and brush cytology were inconclusive. The case was subsequently rediscussed at a multidisciplinary meeting and, given the persistent suspicion of malignancy, surgical treatment was recommended.

**Fig. 3.**
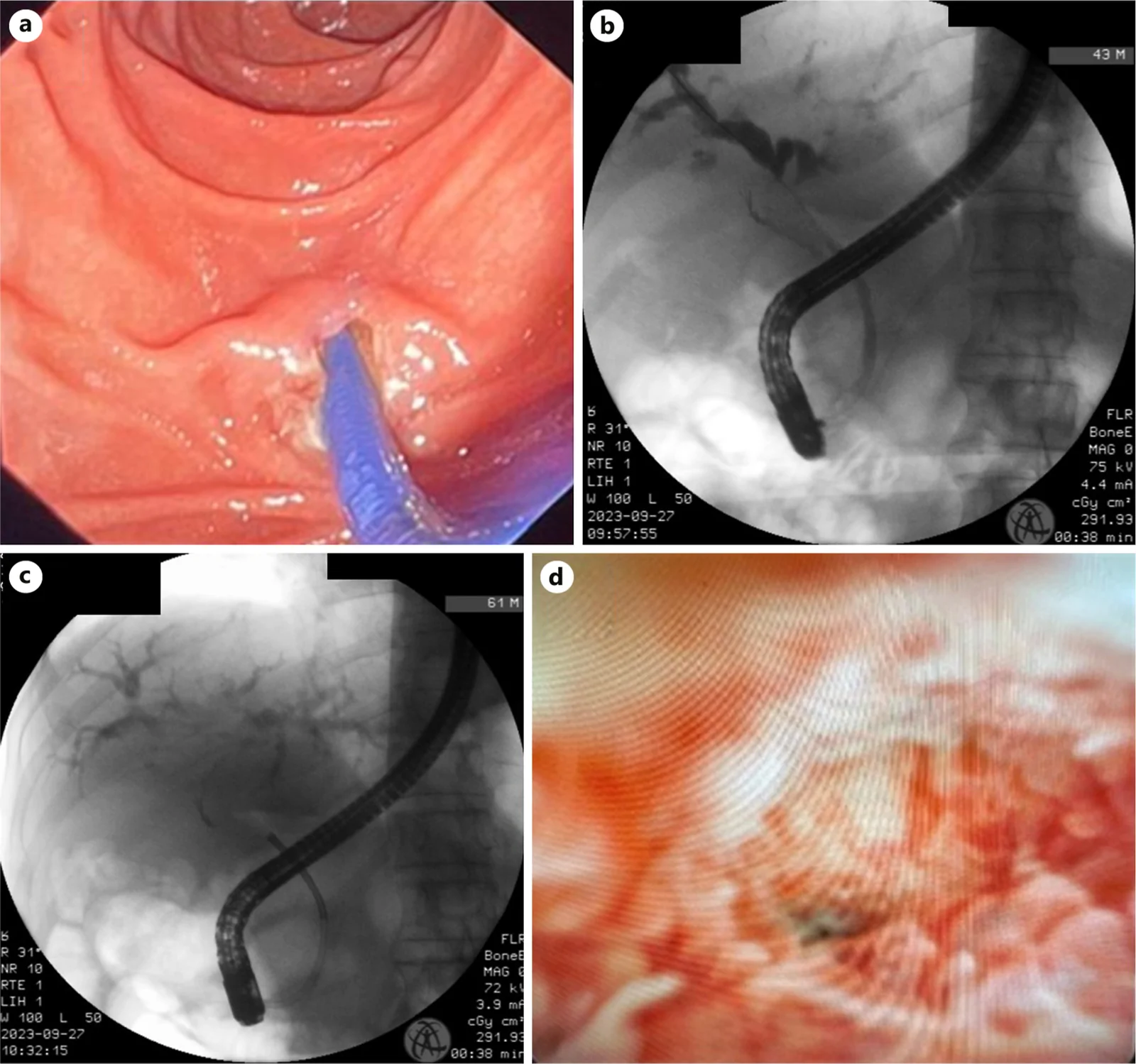
ERCP with septotomy for biliary access associated with the double guidewire technique (**a**); cholangiogram showed a moderate dilatation of the intrahepatic bile ducts with abrupt 3-cm long interruption in the hilar region (**b**, **c**); cholangioscopy showed an irregular mucosa with amorphous appearance, characterized by tortuous neovessels, marked friability and irregular nodularities in the proximal third of the main bile duct; in the proximal portion of these alterations, a stenosis was identified (**d**).

The patient underwent right hepatectomy extended to segment 1 with extrahepatic bile duct resection and Roux-en-Y hepaticojejunostomy. The postoperative course was complicated by a grade B biliary fistula and a biloma, successfully managed with percutaneous drainage and antibiotic therapy.

Histopathological examination demonstrated marked fibroinflammatory thickening of both intrahepatic and extrahepatic bile ducts, characterized by storiform fibrosis and dense lymphoplasmacytic infiltrates rich in plasma cells. Immunohistochemical analysis showed an IgG4/IgG ratio of approximately 55%, supporting the diagnosis of IgG4-SC in the appropriate clinicopathological context. No evidence of carcinoma was identified (Fig. 4).

**Fig. 4.**
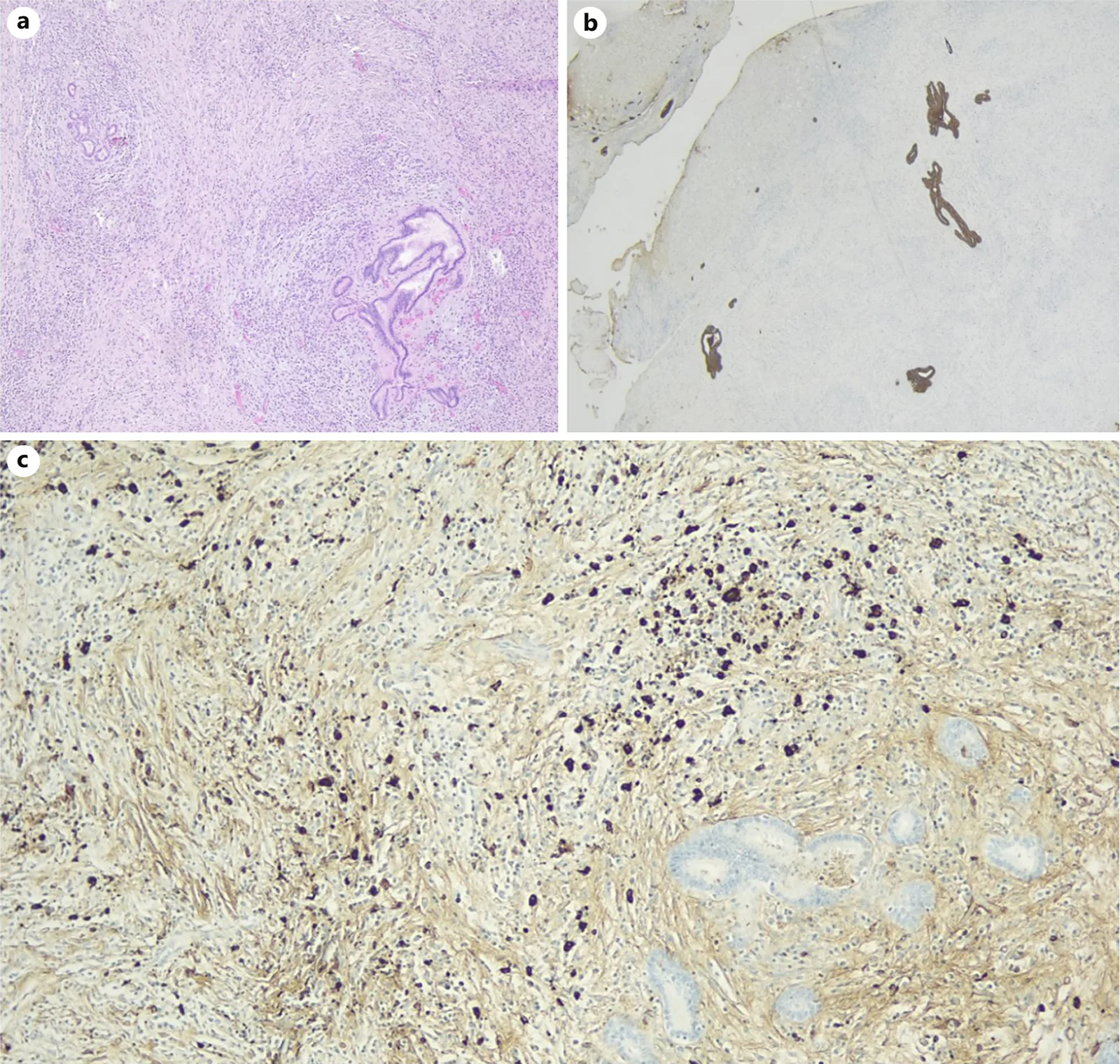
Microscopic study with hematoxylin and eosin staining showed a large and small extrahepatic and intrahepatic bile ducts with thickening at the expense of fibrous tissue with a storiform pattern and mixed-type inflammatory infiltrate, predominantly mononuclear, with numerous plasma cells (**a**); no carcinoma identified, confirmed by immunohistochemical study with CK7 (**b**); the IgG4/IgG ratio is around 0.55 (**c**).

At 1-month postoperative follow-up, the patient was clinically well, reporting only occasional nonspecific abdominal discomfort. Laboratory evaluation demonstrated persistent cholestatic liver enzyme elevation (ALP 609 U/L, GGT 1,141 U/L) despite improvement in bilirubin levels, and serum IgG4 was elevated at 166 mg/dL (normal <135). Following discussion with the Hepatology Department, corticosteroid therapy was initiated with prednisolone at a dose of 0.5 mg/kg/day. After 1 month of treatment, liver biochemistry was improving (ALP 463 U/L, GGT 904 U/L) and serum IgG4 levels had decreased substantially from 166 mg/dL to 72 mg/dL. A sustained biochemical response was observed thereafter, with progressive normalization of cholestatic liver enzymes throughout follow-up, as shown in Table 6, allowing gradual reduction of prednisolone. At 1-year follow-up, the patient remained asymptomatic, with near-complete normalization of liver biochemistry (ALP 67 U/L, GGT 80 U/L) while receiving maintenance prednisolone at a dose of 5 mg/day.

**Table 6.** Evolution of liver biochemistry and serum IgG4 levels following initiation of corticosteroid therapy

​	Before corticosteroid therapy	After corticosteroid therapy				
		1 month	2 months	3 months	6 months	12 months
AST, U/L	59	55	52	54	44	35
ALT, U/L	177	154	144	121	51	42
ALP, U/L	609	463	324	176	165	67
GGT, U/L	1,141	904	691	400	170	80
Total bilirubin, mg/dL	1.2	1.0	1.1	0.8	0.9	0.7
Immunoglobulin G4, mg/dL	166	72	69	ND	ND	111

AST, aspartate aminotransferase; ALT, alanine aminotransferase; ALP, alkaline phosphatase; GGT, gamma-glutamyl transferase; ND, not determined.

Reference values: AST <50 U/L, ALT <50 U/L, ALP 30–120 U/L, GGT <55 U/L, total bilirubin 0.3–1.2 mg/dL, IgG4 <135 mg/dL.

## Discussion

IgG4-SC is a rare fibroinflammatory disease that frequently poses a significant diagnostic challenge due to its ability to closely mimic CCA in clinical presentation, laboratory findings, and imaging characteristics [2, 6]. Distinguishing between these entities is critical, as their therapeutic approach differ substantially; IgG4-SC typically responds well to corticosteroid therapy, whereas CCA requires oncologic resection when feasible.

Both conditions commonly present with progressive obstructive jaundice and cholestatic liver enzyme abnormalities [8]. However, certain clinical features may help the differential diagnosis. Jaundice associated with CCA is often painless, whereas IgG4-SC more frequently presents with abdominal discomfort [10]. Constitutional symptoms such as weight loss and asthenia are more suggestive of malignancy but may also occur in advanced inflammatory disease [10]. Importantly, the presence of other IgG4-related manifestations, such as autoimmune pancreatitis, sialadenitis or dacryoadenitis, may facilitate the differential diagnosis and strongly support the diagnosis of IgG4-SC. In the present case, no such manifestations were identified. The patient did have a history of AL-type amyloidosis involving the gastrointestinal tract; however, no established pathogenic relationship between IgG4-SC and AL amyloidosis has been reported. While rare cases of AA amyloidosis have been described in association with IgG4-related disease [11], the coexistence of AL amyloidosis in our patient was most likely incidental.

An additional noteworthy aspect of this case is the patient’s occupational history in railway track maintenance. Previous studies have reported a higher prevalence of blue-collar occupations among patients with IgG4-related disease, although the clinical significance of this association remains uncertain [12].

Serological markers provide supportive but not definitive evidence. Elevated serum IgG4 levels are highly suggestive of IgG4-SC, particularly when exceeding twice the upper limit of normal; nevertheless, up to one-third of patients may have normal concentrations [4, 7]. Conversely, tumor markers such as CA 19-9 lack specificity and may be elevated in benign conditions associated with cholestasis or inflammation, including IgG4-SC [2]. Interpretation of CA 19-9 levels in patients with obstructive jaundice should therefore be performed cautiously, as cholestasis itself may contribute to elevated values. Several authors have proposed correction models incorporating bilirubin levels to improve diagnostic accuracy, although these approaches have not yet been incorporated into current clinical practice guidelines [13].

Imaging evaluation plays a pivotal role in the assessment of biliary strictures; however, there is substantial overlap between IgG4-SC and CCA. Both conditions may present with concentric or irregular thickening of the bile duct wall and biliary strictures on cross-sectional CT imaging [6]. CCA typically demonstrates heterogeneous and relatively hypoenhancing lesions during the arterial phase, followed by progressive enhancement in the portal venous and delayed phases. Nevertheless, IgG4-SC may also exhibit gradual delayed enhancement, limiting the specificity of this feature [14, 15]. On magnetic resonance imaging, IgG4-SC more commonly shows a homogeneous and diffuse T2-weighted signal, occasionally accompanied by a peripheral “halo sign” described in some studies, although this finding is not pathognomonic. In contrast, CCA usually presents with more focal diffusion restriction on diffusion-weighted imaging, irregular margins, and a greater propensity for vascular encasement and lymph node involvement, features that may favor a malignant etiology [14, 15].

Endoscopic evaluation with cholangioscopy can aid in the differential diagnosis of biliary strictures by enabling direct visualization of the biliary mucosa. Endoscopic findings may vary according to disease stage; in the active phase of inflammatory disease, mucosal erythema, ulceration, and fibrinous exudate may be observed, whereas chronic disease is more often associated with fibrotic scarring and pseudodiverticulum formation. In contrast, CCA typically presents with an irregular, friable mucosa and dilated, tortuous vessels. However, no endoscopic pattern is pathognomonic and careful interpretation in conjunction with clinical, laboratory, and imaging findings is essential [16]. An additional advantage of cholangioscopy is the ability to obtain targeted endobiliary biopsies, which may improve diagnostic yield in indeterminate biliary strictures [17]. Nevertheless, obtaining representative tissue may remain challenging due to the dense subepithelial fibrosis characteristic of IgG4-SC [2]. Endoscopic ultrasound-guided biopsy represents a complementary approach, allowing targeted sampling of suspicious lesions across multiple tissue layers and potentially providing more representative material for histopathological evaluation [18].

Current guidelines suggest that when IgG4-SC is strongly suspected and malignancy has been reasonably excluded, a trial of corticosteroid therapy may serve both diagnostic and therapeutic purposes, with rapid clinical and biochemical improvement supporting the diagnosis [19, 20]. In the present case, serum IgG4 levels were not measured during the initial diagnostic workup. However, several features strongly favored a malignant etiology, including an isolated hilar stricture, suspicious cholangioscopic findings, persistently elevated CA 19-9 levels following biliary drainage, and the lack of extra-biliary manifestations suggestive of IgG4-related disease. Taken together, these findings supported prioritization of oncologic resection. In retrospect, earlier assessment of serum IgG4 might have broadened the differential diagnosis; however, an isolated elevation would not have been sufficient to definitively exclude CCA.

This case illustrates the persistent diagnostic challenge posed by isolated IgG4-SC presenting as a solitary hilar biliary stricture. Despite advances in imaging, endoscopic assessment, and tissue sampling, reliable preoperative distinction from CCA remains difficult. Indeed, surgical series have shown that approximately 15% of patients undergoing resection for presumed perihilar CCA are ultimately found to have benign disease, with IgG4-associated cholangitis accounting for a substantial proportion of these cases [21]. Greater awareness of this entity, routine consideration of serum IgG4 testing, and multidisciplinary evaluation may improve diagnostic accuracy and help avoid unnecessary major hepatobiliary resections. Nevertheless, when malignancy cannot be confidently excluded, particularly in patients with potentially resectable disease, surgical resection remains the most appropriate and safest strategy, with definitive diagnosis sometimes only achievable on histopathological examination of the resected specimen.

## Acknowledgments

Generative artificial intelligence (AI) was used during the preparation of this manuscript for language editing, text refinement, and assistance with the identification and verification of relevant literature. ChatGPT (OpenAI) was used as an AI-assisted tool [22]. All AI-generated or AI-assisted content was critically reviewed and independently verified by the authors against the original clinical data and source publications. The authors take full responsibility for the accuracy, originality, and integrity of the final manuscript. No AI tool was used to generate or interpret patient data, clinical findings, diagnostic results, or treatment decisions.

## Statement of Ethics

This study was performed in accordance with the Declaration of Helsinki. Ethics approval was not required in accordance with national guidelines. Written informed consent was obtained from the individual for publication of the details of their medical case and any accompanying images. The CARE Checklist has been completed by the authors for this case report, attached as online supplementary material (for all online suppl. material, see https://doi.org/10.1159/crg/abwag011).

## Conflict of Interest Statement

The authors have no conflicts of interest to declare.

## Funding Sources

This study was not supported by any sponsor or funder.

## Author Contributions

T.A. case conception, clinical data collection, and manuscript drafting. D.F. clinical data collection and manuscript drafting. J.M. histopathological evaluation and detailed description of microscopic findings. M.J.A. critical manuscript review and contribution to clinical interpretation. C.M. literature review and manuscript editing. M.S. participation in multidisciplinary discussion and manuscript review. J.G.T. supervision, scientific guidance, and final manuscript approval. All authors read and approved the final version.

## Data Availability

The data supporting this article consist solely of the patient’s clinical and imaging information presented in the case report. These data are not publicly available due to patient confidentiality. Additional information may be provided by the corresponding author upon reasonable request and with patient authorization. Publication Ethics and Editorial Policies | Karger Publishers.
